# Supplementary material for: Design and characterization of a protein fold switching network
Source: Nat Commun. 2023 Jan 26;14:431. doi: 10.1038/s41467-023-36065-3 (PMC9879998; doi:10.1038/s41467-023-36065-3)
Supplement: Supplementary file 1 — Supplementary Information [file 41467_2023_36065_MOESM1_ESM.pdf]

Supplemental Table 1. Fold, stability, and binding properties for each designed protein.

Mutant	Fold	$\Delta G_{folding}$ at 25˚C	Binding ligand	K_I and or K_D
SI	S	-8.5 kcal/mol	Protease	0.2μM
S_{a1}I	S	-5.3 kcal/mol	Protease	0.05μM
S_{a1}	S	-5.3 kcal/mol	---	
A_1	A	-4 kcal/mol	---	
S_{a2}I	S	-5.3 kcal/mol	Protease/HSA	0.05μM/100μM
A_2	A	-4 kcal/mol	HSA	≤ 1μM
S_{b1}	B	-1.1 kcal/mol	---	
B_1	B	-4 kcal/mol	---	
S_{b2}	S	-4 kcal/mol	---	
B_2	unfolded	>2 kcal/mol	---	
S'I	S	-8.5 kcal/mol	Protease	0.06μM
S_{b3}I	S	-3.5 kcal/mol	Protease/IgG	0.05μM/10μM
S_{b3}	S	-3.5 kcal/mol	IgG	10μM
B_3	B	-1.2 kcal/mol	IgG	≤ 1μM
S_{b4}	Mixed S/B	-4 kcal/mol[1]	IgG	≤ 1μM
B_4	B	-4.1 kcal/mol	IgG	≤ 1μM
S_{b5}	B	-5 kcal/mol	IgG	10μM

[1] S_{b4} populates both S- and B-folds in approximately equal mixture. Thus, $\Delta G_{B\text{-fold/S-fold}}$ is ~ 0 kcal/mol. This results from the fact that the stabilities for both B- and S-fold species relative to the unfolded state (determined by CD melting) are almost identical (i.e., $\Delta G_{folding}$ ~ -4 kcal/mol).

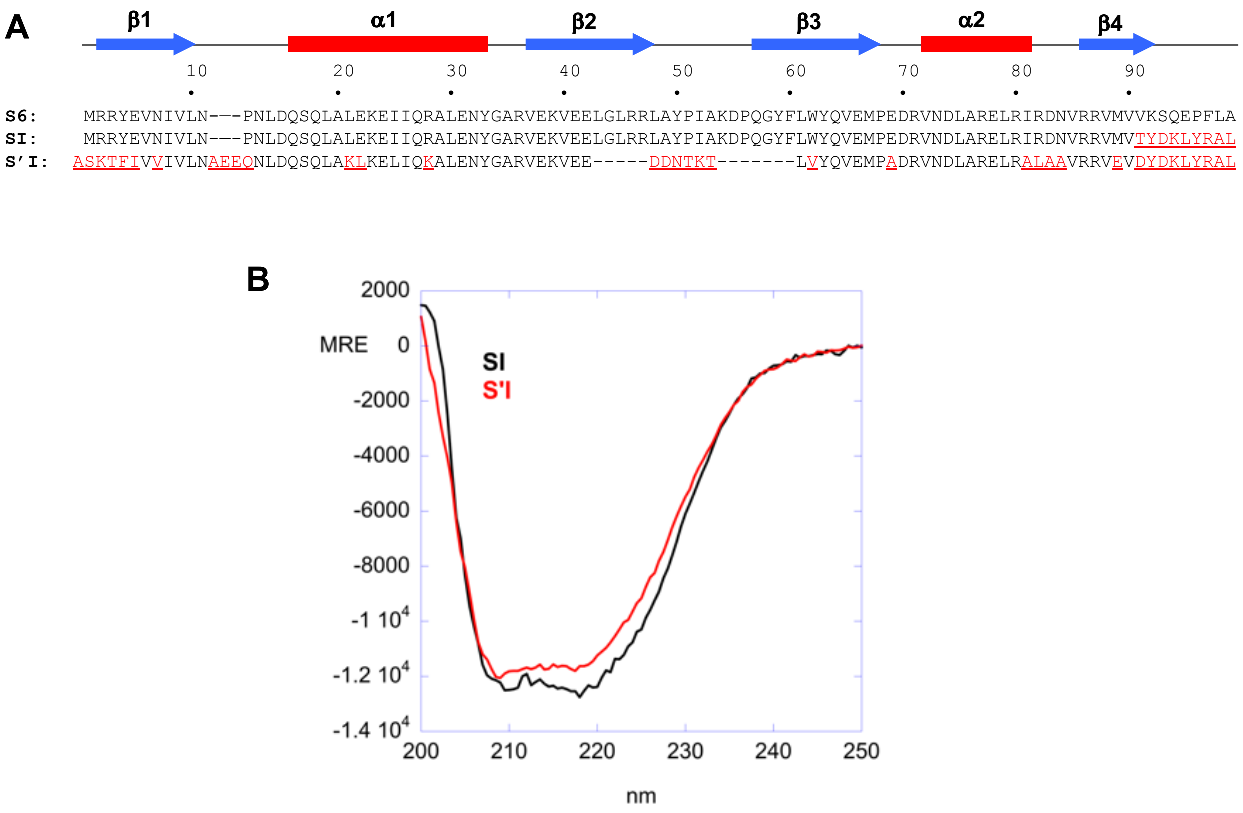

Supplemental Figure 1: Sequence comparison of S6, SI, and S’I. The secondary structure for the parent S6 fold is shown above the alignment. Mutations are shown by red text. CD spectra are compared in panel B. Source data are provided as a Source Data file.

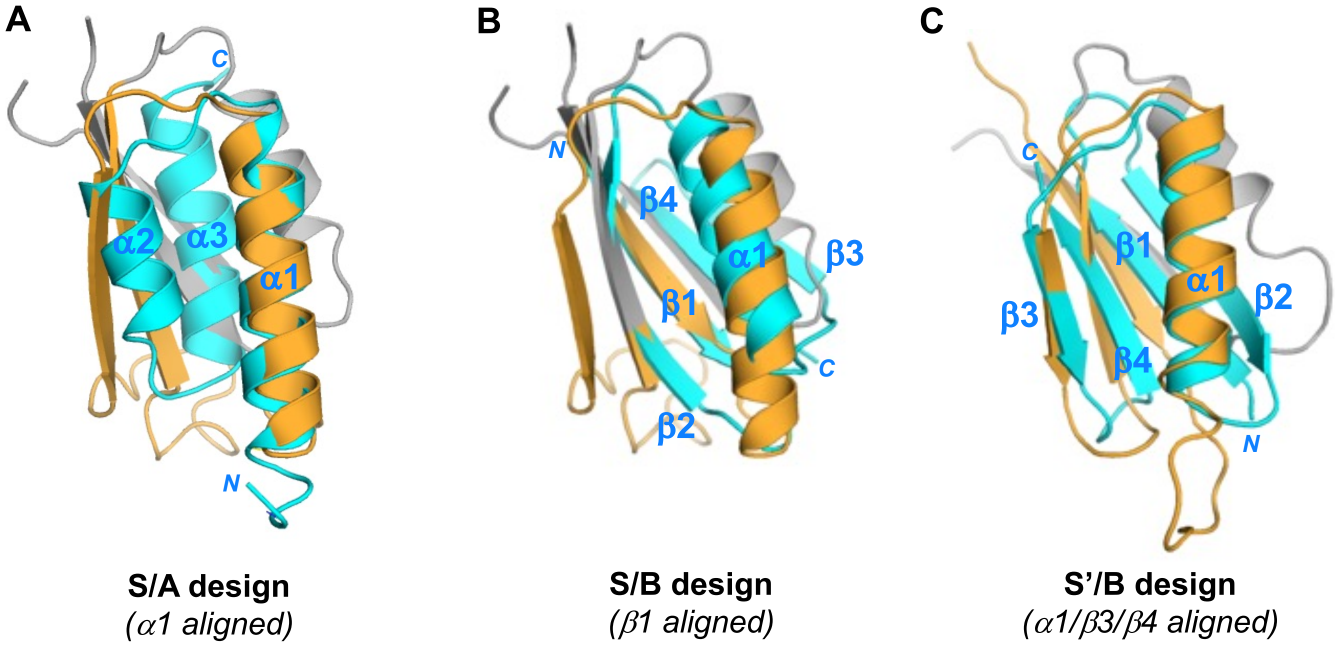

Supplemental Figure 2: Topological alignment of A and B-folds with S-folds. (A) The embedded A-fold occupies amino acid positions 11-66 with 46% identity in the aligned regions for the parent sequences. $\alpha 1$(S) mostly coincides with $\alpha 1$(A). $\beta 2$(S) becomes $\alpha 2$(A), and the long $\beta 2$-$\beta 3$ turn and first half of $\beta 3$(S) becomes $\alpha 3$(A). $\beta 1$ and $\alpha 2$-$\beta 4$ of the S-fold are outside of the overlap region. (B) The embedded B-fold occupies amino acid positions 4-59 with 9% identity in the aligned regions for the parent sequences. $\beta 1$(S) mostly coincides with $\beta 1$(B). The first half of $\alpha 1$(S) becomes $\beta 2$(B) and the second half of $\alpha 1$(S) becomes the first half of $\alpha 1$(B). The $\beta 2$ strand of S becomes the second half of $\alpha 1$(B), a turn, and the first part of $\beta 3$(B). The long $\beta 2$-$\beta 3$(S) turn and the first part of $\beta 3$(S) become the second part of $\beta 3$ and $\beta 4$ of B. The second half of $\beta 3$ and $\alpha 2$-$\beta 4$ of the S-fold are outside of the overlap region. (C) The embedded B-fold occupies amino acid positions 1-56 with 16% identity in the aligned regions for the parent sequences (excluding the 3 residue insertion and 12 residue deletion). The $\beta 1$–strand is the same in both folds but changes orientation, the long turn between $\beta 1$ and $\alpha 1$ in S_{b3} becomes $\beta 2$ of the B-fold, and the $\alpha 1$–$\beta 2$–$\beta 3$ of S_{b3} maintains the same topology in both folds. The $\alpha 2$-helix and the $\beta 4$–strand of S_{b3} are outside the overlap region.

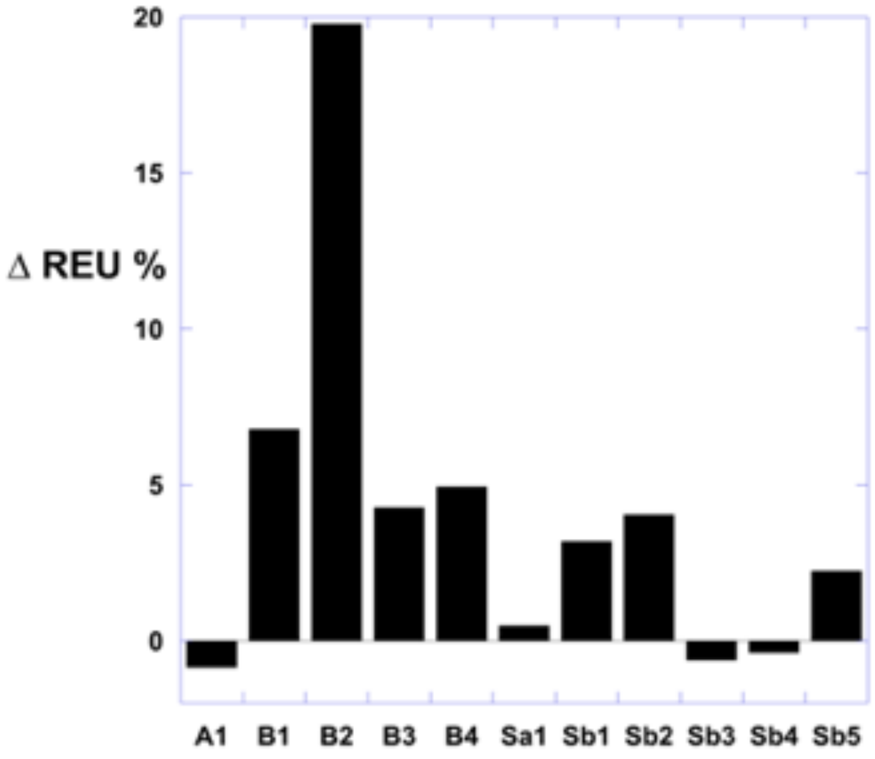

Supplemental Figure 3: The percent change in Rosetta Energy Units (REU) between the parent protein and the designed switch protein is plotted. The computational design of A_1 was compared to the relaxed structure of a highly stable A-fold [2]. The designs of B_1, B_2, B_3, and B_4 were compared to a highly stable B-fold [3]. The designs of S_{a1}, S_{b1}, S_{b2}, S_{b3}, S_{b4}, and S_{b5} were compared to a highly stable S-fold [4]. All designed proteins have relatively small changes in %ΔREU except for B_2. The 20% increase in REU for B_2 is consistent with its low stability.

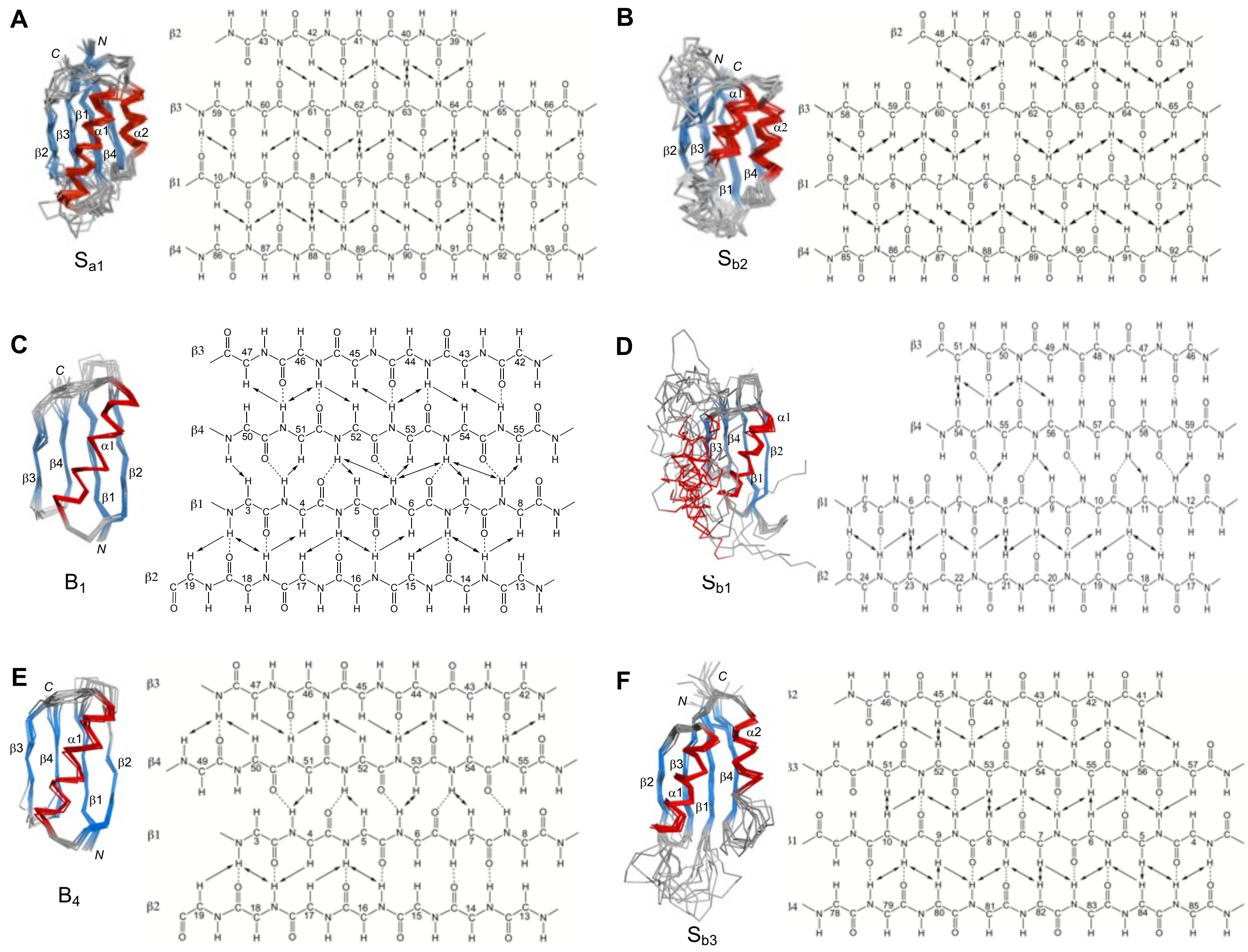

Supplemental Figure 4: Summary of long-range backbone NOEs observed for β-sheets in designed proteins.

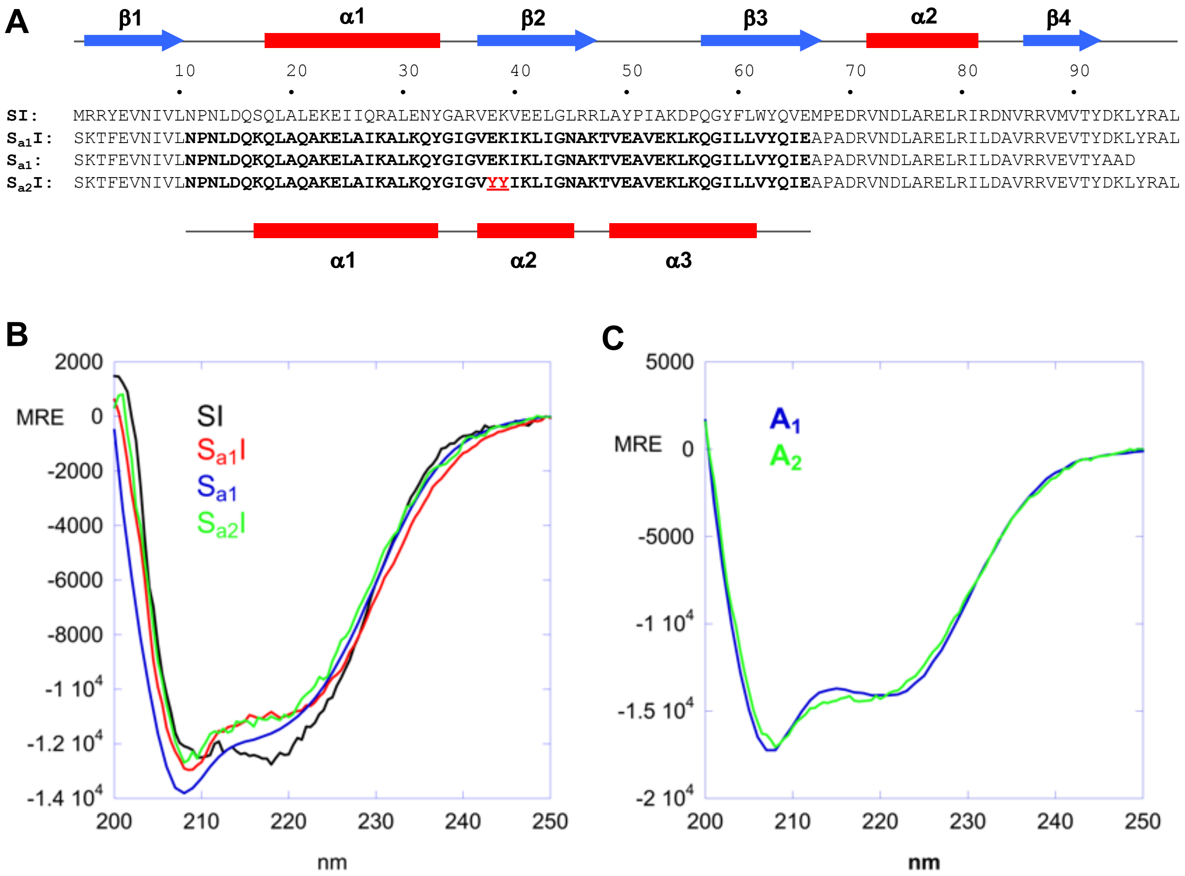

Supplemental Figure 5: Sequence comparison of SI, S_{a1}, S_{a2}, and S_{a2}I. The secondary structures for the parent S6 and A folds are shown above and below the alignment, respectively. Mutations are shown by red text. CD spectra are compared in panels B and C. Source data are provided as a Source Data file.

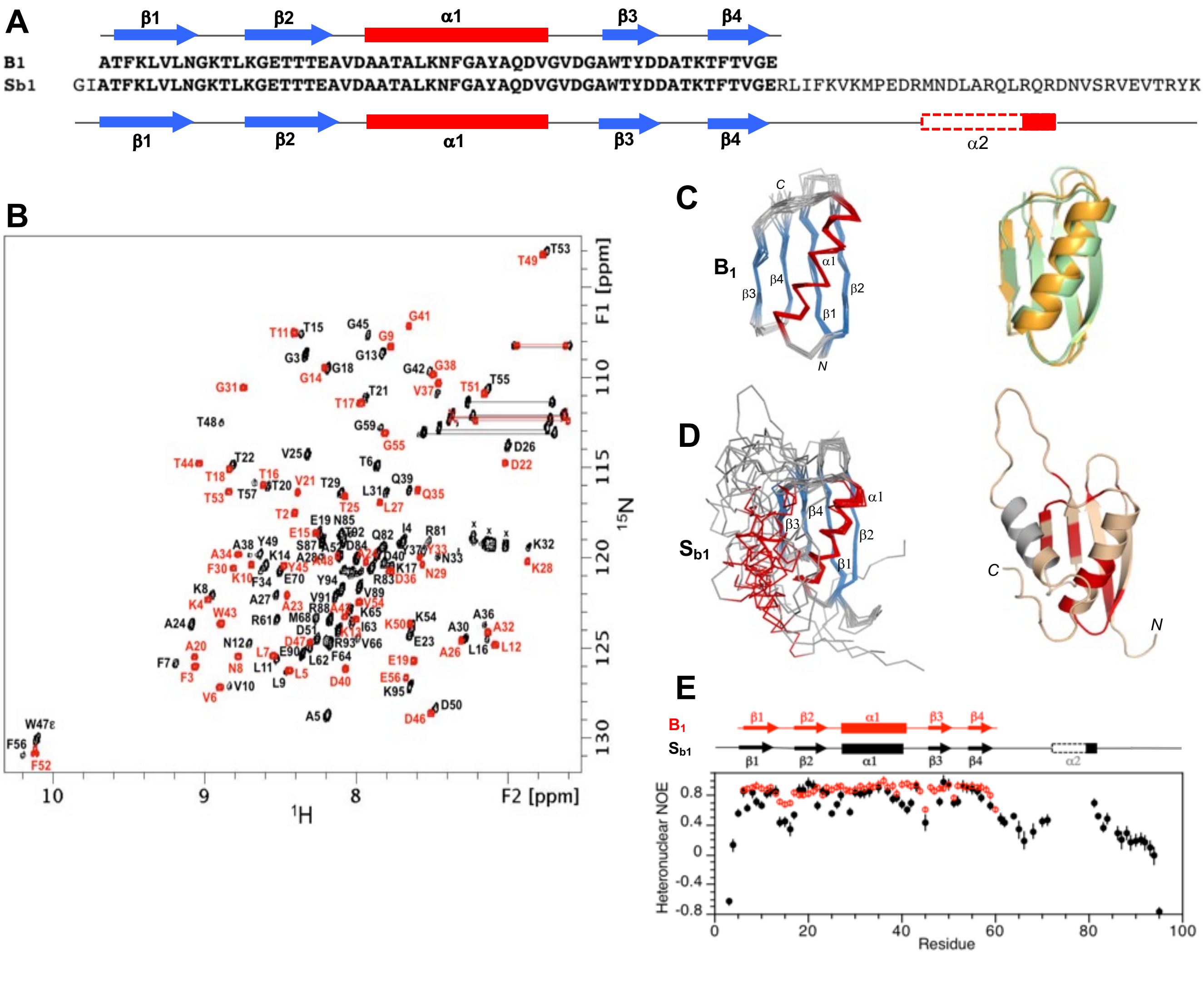

Supplemental Figure 6: Structure and dynamics of B_1 and S_{b1} - S_{b1} has a B-fold. (A) Sequence alignment of B_1 and S_{b1}, which are 100% identical over the B-region. (B) Overlaid two dimensional ^{1}H-^{15}N HSQC spectra of S_{b1} (black) and B_1 (red) with backbone amide assignments. Spectra were recorded at 10°C. (C) Ensemble of 10 lowest energy CS-Rosetta structures for B_1 (left panel). Superposition of the B_1 structure (green) with the parent GB1 fold (orange) (right panel). (D) Ensemble of 10 lowest energy CS-Rosetta structures for S_{b1} using chemical shift, NOE, and PRE restraints (left panel). Cartoon representation of model 1 from the ensemble (right panel). Values of $\Delta\delta_{total}$>0.1 ppm from Fig. S7A are mapped onto the structure (red). Unassigned residues in the putative α2-helix are in gray. (E) Plot of {^{1}H}-^{15}N steady state heteronuclear NOE values at 600 MHz versus residue for B_1 (red) and for S_{b1} (black). Each set of heteronuclear NOEs was obtained from a single experiment. Errors were estimated based on the measured background noise level.

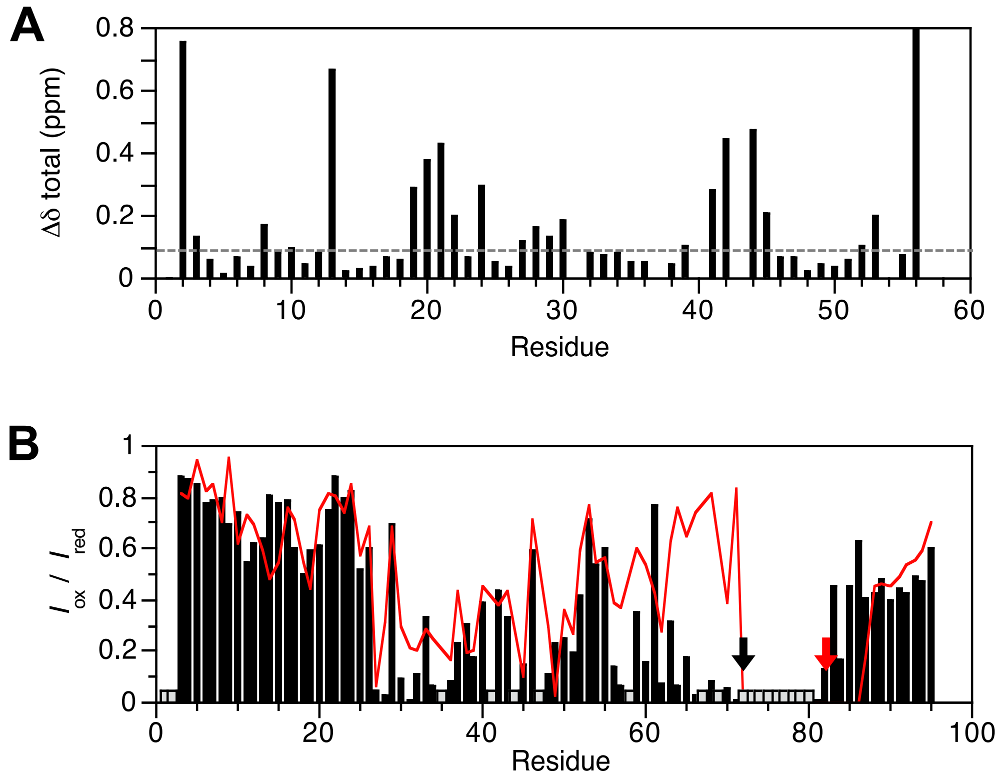

Supplemental Figure 7: Chemical shift perturbation (CSP) and PRE profiles for S_{b1}. (A) Plot of CSPs between S_{b1} and B_1 for backbone amides in the 56 amino acid identical region. Residue numbering is for B_1. (B) Plot of I_{ox}/I_{red} versus residue for S_{b1}-R72C-MTSL (black) and S_{b1}-R83C-MTSL (red). Similar PRE profiles are observed in the B-folded part of the structure when the spin label is attached to the N- or C-terminus of the exchange-broadened region, which corresponds with α2 in S6. Gray columns indicate unassigned residues or prolines. The positions of the spin labels are indicated with arrows.

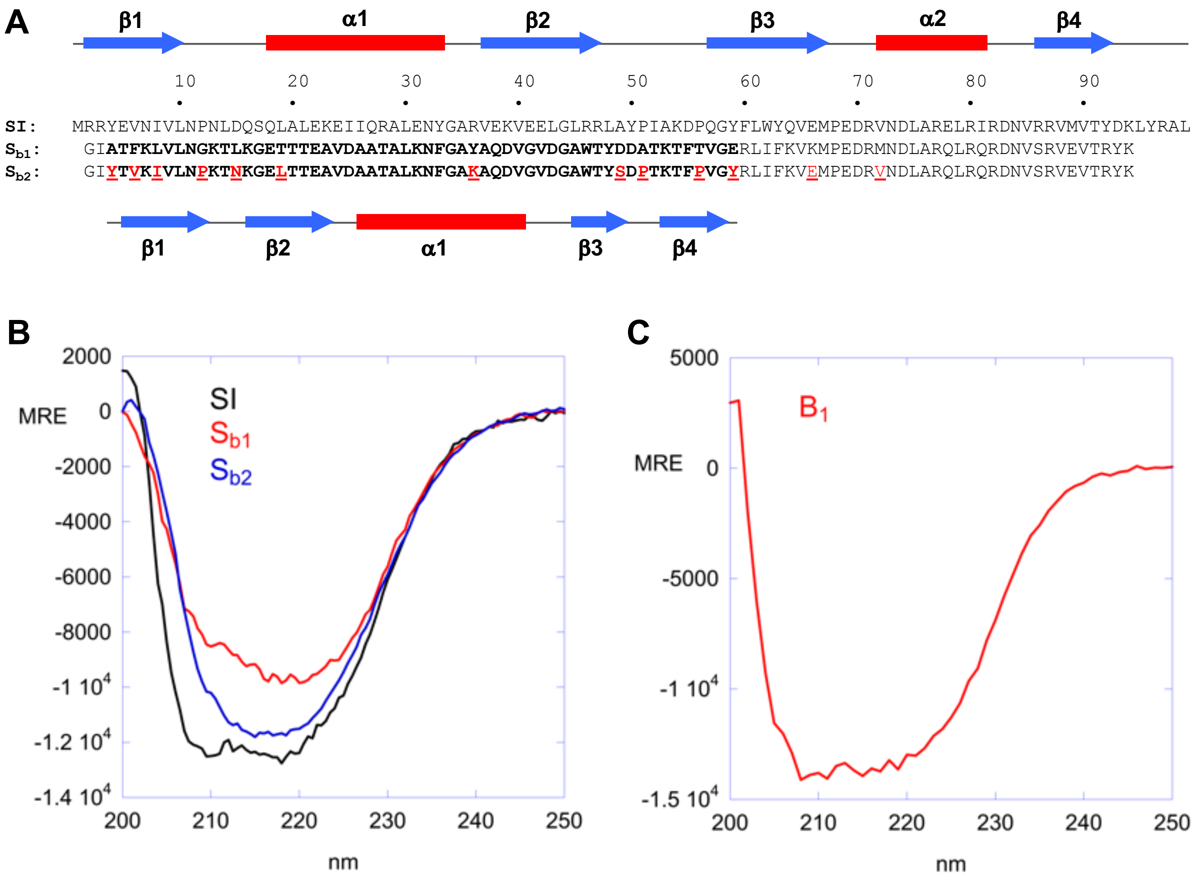

Supplemental Figure 8: Sequence comparison of SI, S_{b1}, and S_{b2}. The secondary structures for the parent S6 and B folds are shown above and below the alignment, respectively. Mutations are shown by red text. CD spectra are compared in panels B and C. Source data are provided as a Source Data file.

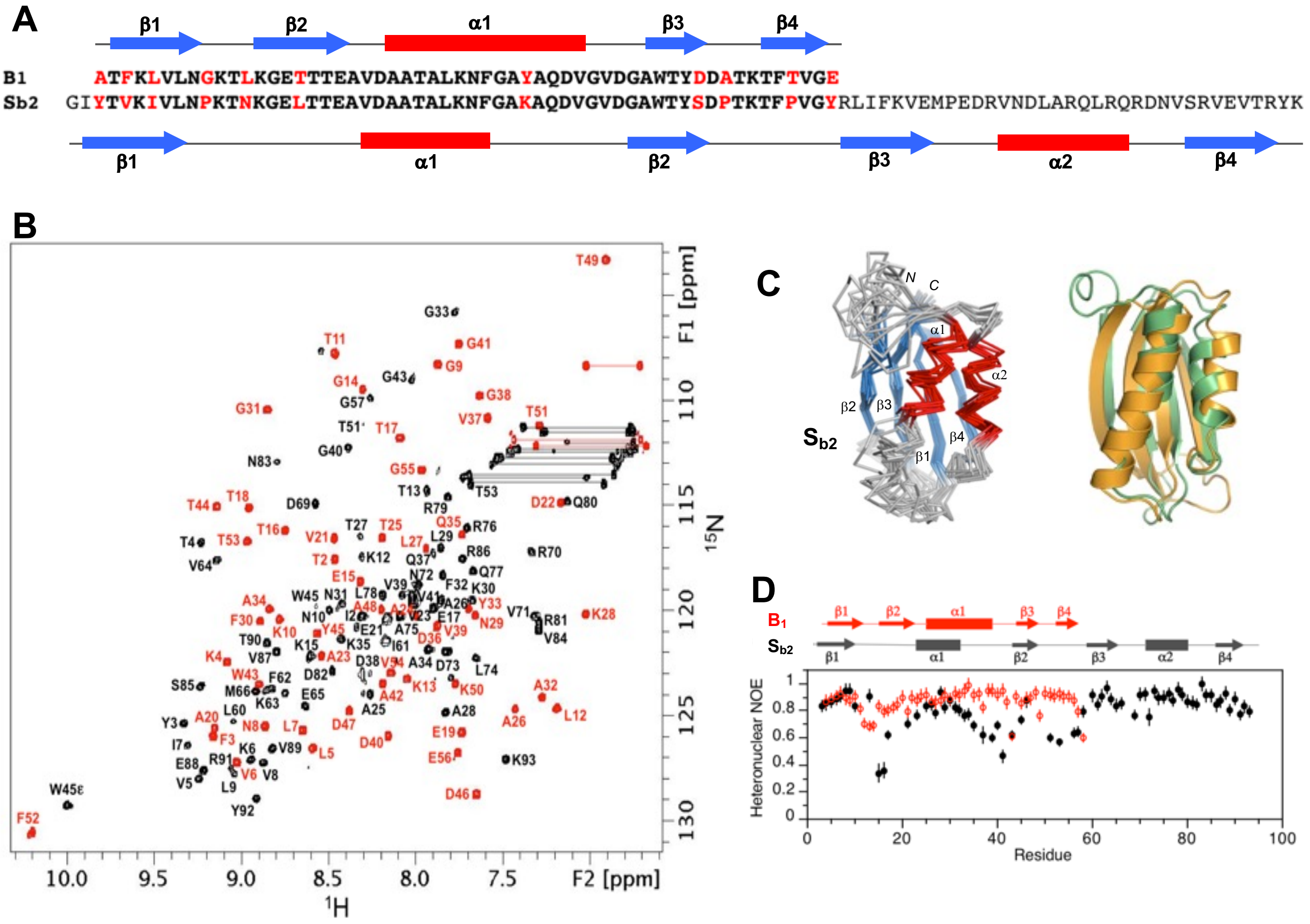

Supplemental Figure 9: Structure and dynamics of S_{b2} – S_{b2} has an S-fold. (A) Sequence alignment of S_{b2} and B_1. The 11 amino acid differences are shown in red. (B) Overlaid two dimensional ^{1}H-^{15}N HSQC spectra of S_{b2} (black) and B_1 (red) with backbone amide assignments. Spectra were recorded at 25ºC. (C) Ensemble of 10 lowest energy CS-Rosetta structures for S_{b2} (left panel). Superposition of S_{b2} (green) with the parent S6 fold (orange) (right panel). (D) Plot of {^{1}H}-^{15}N steady state heteronuclear NOE values at 600 MHz versus residue for S_{b2} (black). Values are compared with B_1, which has 80% sequence identity in the corresponding region. Each set of heteronuclear NOEs was obtained from a single experiment. Errors were estimated based on the measured background noise level.

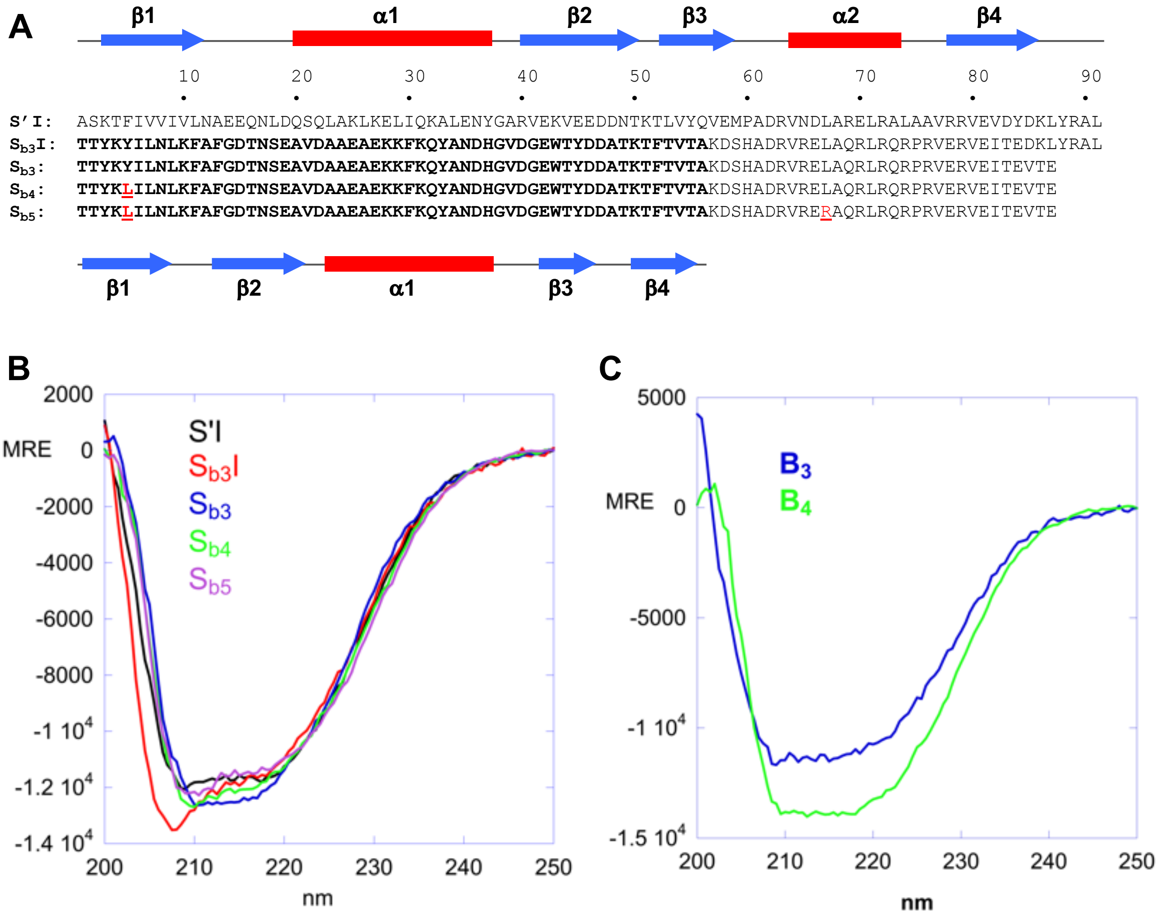

Supplemental Figure 10: Sequence comparison of S'I, S_{b3}, S_{b4}, and S_{b5}. The secondary structures for the parent S6 and B folds are shown above and below the alignment, respectively. Mutations are shown by red text. CD spectra are compared in panels B and C. Source data are provided as a Source Data file.

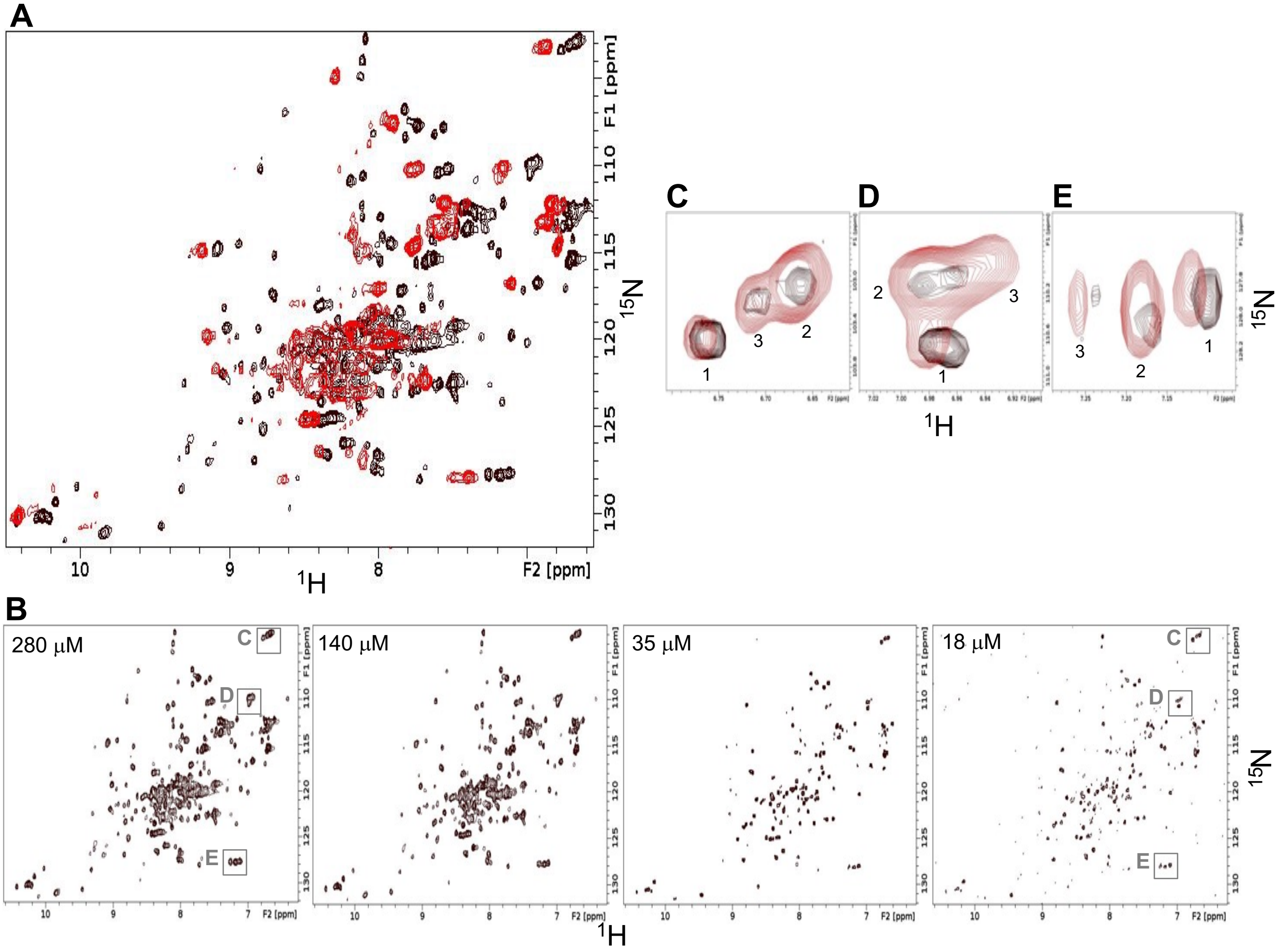

Supplemental Figure 11: Temperature and concentration dependence of B_3. (A) Overlaid two dimensional ^{1}H-^{15}N HSQC spectra of B_3 at 25°C (red) and at 5°C (black). The protein concentration in both spectra is 280 μM. Broadened peaks at 25°C are consistent with low stability. (B) HSQC spectra of B_3 recorded at different concentrations as indicated. All spectra were acquired at 5°C. (C, D, E) Expanded regions from the overlaid concentration dependent spectra of B_3 at 18 μM (black) and 280 μM (red). Regions are as indicated in the leftmost and rightmost panels in (B). The peaks in (C, D, E) are labeled 1, 2 and 3, corresponding to three main concentration-dependent species. At 18 μM, the putatively monomeric species 1 is dominant, while at 280 μM the putatively dimeric species 2 is dominant.

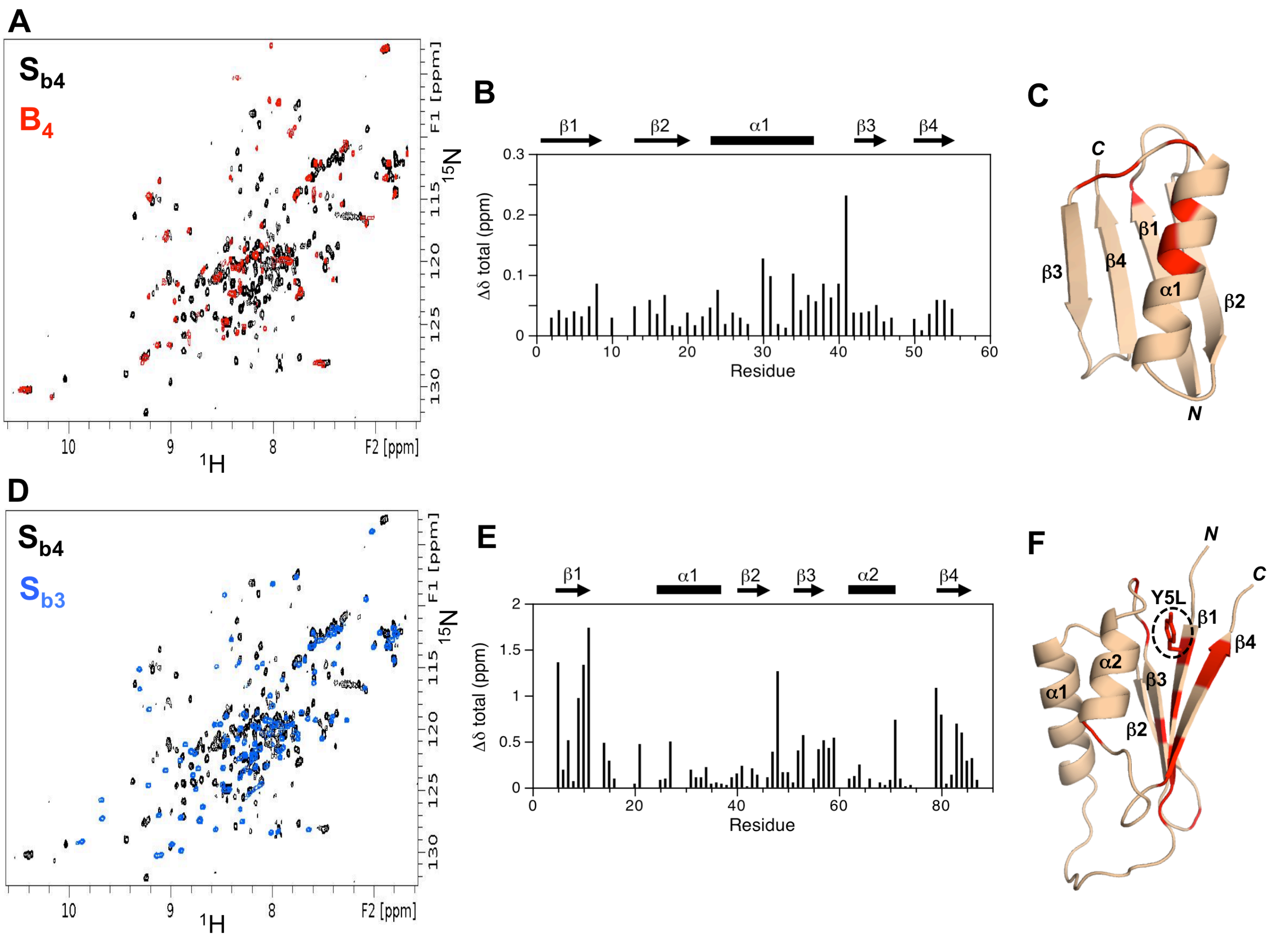

Supplemental Figure 12: Chemical shift comparisons for S- and B-states of S_{b4} with S_{b3} and B_4, respectively. (**A**) Overlaid two-dimensional ^{1}H-^{15}N HSQC spectra of S_{b4} (black) and B_4 (red). (**B**) Backbone amide chemical shift differences ($\Delta\delta_{total}$) between the B-state of S_{b4} and B_4 at 25°C versus residue. The secondary structure of B_4 is shown above the plot. (**C**) Residues with values of $\Delta\delta_{total}$ > 0.08 ppm (red) are mapped onto the structure of B_4. (**D**) Overlaid two-dimensional ^{1}H-^{15}N HSQC spectra of S_{b4} (black) and S_{b3} (blue). (**E**) Backbone amide chemical shift differences between the S-state of S_{b4} and S_{b3} at 25°C versus residue. The secondary structure of S_{b3} is shown above the plot. (**F**) Residues with values of $\Delta\delta_{total}$ > 0.5 ppm (red) are mapped onto the structure of S_{b3}. The position of the single amino acid difference between S_{b3} and S_{b4}, Y5 in S_{b3} and L5 in S_{b4}, is highlighted.

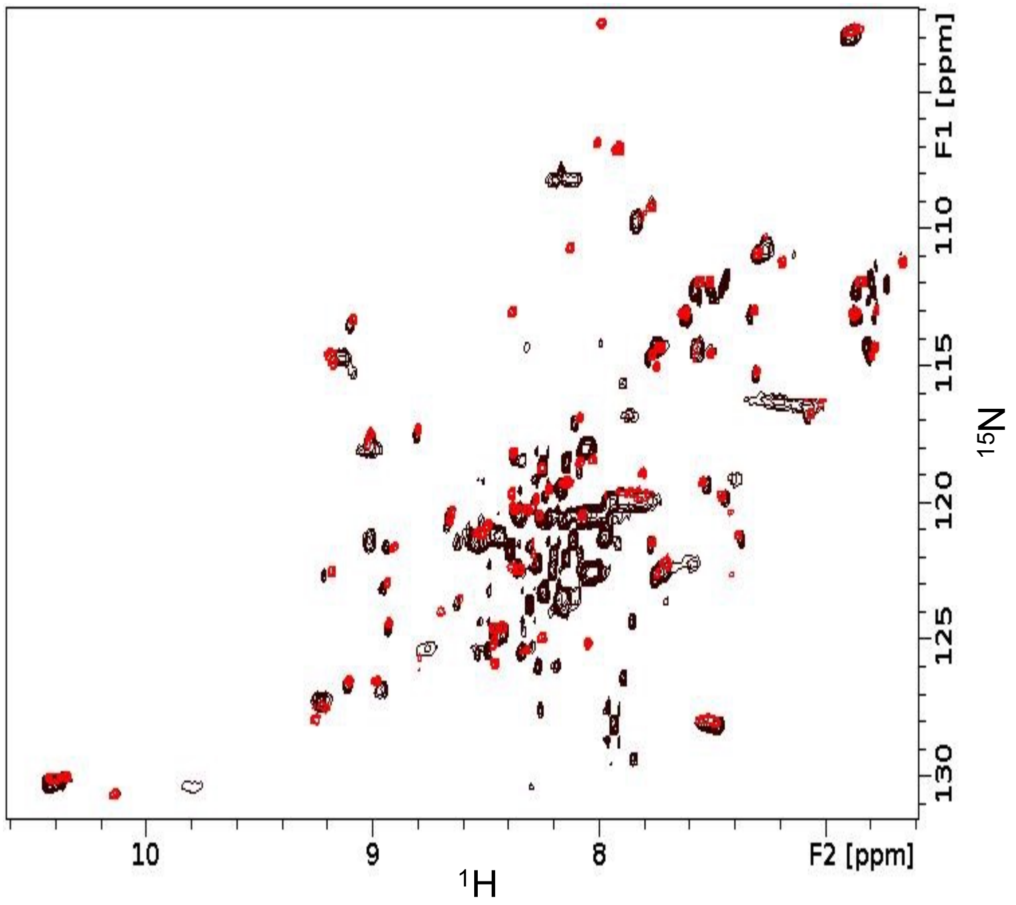

Supplemental Figure 13: Two dimensional ^{1}H-^{15}N HSQC spectrum of S_{b5} (black) compared with B_4 (red). Both spectra were acquired at 25°C. The significant overlap of folded signals in the two spectra indicates that S_{b5} has a B-fold with a disordered C-terminal tail.

Supplemental description of strucures

Structure of B_1**.** The NMR structure consists of four β-strands defined by residues 2-9 (β1), residues 13-20 (β2), residues 42-46 (β3), and residues 50-55 (β4) and one α-helix from residues 23-37 (**Fig. S6B,C, Table 1**). The NOE pattern between main chain protons agrees well with a B-fold (**Fig. S4C).** Steady-state $\{^1H\}$-^{15}N heteronuclear NOE values for B_1 are consistent with a well ordered structure, with the exception of the β1-β2, α1-β3, and β3-β4 internal loops (**Fig. S6E**).

Structure of S_{b1}**.** The topology of S_{b1} is not the same as the parent S6 structure. Instead, the 2D ^1H-^{15}N HSQC spectrum of S_{b1} has a pattern similar to that of B_1 (**Fig. S6B)**. NMR assignment of the main chain resonances showed the presence of four β-strands and two α-helices, but the order of the secondary structure elements was ββαββα rather than the βαββαβ arrangement expected for an S-type fold. Initial NMR structures of S_{b1} indicated a B-fold, which was supported by backbone NOE connectivities (**Fig. S4D**), with a mostly disordered C-terminal tail. CS-Rosetta modeled residues 73-83 near the C-terminus as an α2-helix. Of these, amide signals due to residues 73-80 were not detectable in NMR spectra while residues 81-83 were helical based on assigned chemical shifts. Comparison of S_{b1} amide chemical shifts with those of B_1 indicated that most of the perturbations due to the C-terminal 35 amino acid tail were localized in α1, β3, and neighboring regions (**Fig. S7A**). This suggested that the putative α2-helix interacts mostly with the B-fold in these contiguous regions. Mutations R72C and R83C were made at the N- and C-terminal ends of the α2 region in separate samples of S_{b1} and these proteins were derivatized with the stable nitroxide spin label MTSL. Paramagnetic relaxation enhancement (PRE) measurements (**Fig. S7B**) showed significant decreases in amide peak intensity over the α1 and β3 regions for the B-core of S_{b1}, consistent with the chemical shift perturbation data. Furthermore, the PRE intensity profiles were similar regardless of which end of the α2 region the spin label resided. This suggests that docking of the α2 region against the B-folded core of S_{b1} is in exchange between multiple states, providing a plausible explanation for why most of the α2 amide resonances are not detectable. Structures for S_{b1} were re-calculated using additional weak (<20Å) PRE restraints, showing an ensemble with a well-defined B-core that has a putative α2-helix packed against it loosely (**Fig. S6D, Table 2**).

Steady-state {^{1}H}-^{15}N heteronuclear NOE data for S_{b1} were consistent with the structure (**Fig. S6E**). In particular, the C-terminal tail becomes more ordered around the α2 region, although these heteronuclear NOE values (0.4-0.7) are still below those of well-ordered regions (>0.8). Thus, the structure of S_{b1} may be viewed as a transitory state between the S- and B-folds. With two α-helices packed against a 4-stranded β-sheet, S_{b1} has the same overall two-layer α/β-sandwich architecture as the S-fold but differs in the topological arrangement of secondary structures.

Structure of S_{b2}**.** The three dimensional structure of S_{b2} contains four β-strands and two α-helices and has the general features of the parent S-fold (**Fig. S9B,C, Table 2**). The ordered regions in the structure are residues 1-9 (β1), 23-32 (α1), 43-48 (β2), 59-65 (β3), 71-80 (α2), and 86-91 (β4). While the parent S6 protein [http://doi.org/10.2210/pdb1RIS/pdb] and S_{a1} structures are very similar, the S_{b2} structure differs from both in a number of ways despite having the same overall topology. The α1-helix in S_{b2} is shorter, comprising 10 amino acids compared with 17 amino acids in S_{a1}. Also, the β2-strand forms 4 amino acids later in the S_{b2} polypeptide chain than in S_{a1}. The first residue in the β2-strand of S_{b2}, G43, interacts with E65 in the β3-strand. This represents a two-residue shift in the register of hydrogen bonding between β2 and β3 in S_{b2} compared with S_{a1} (**Fig. S4B**). As a result of these differences, the loops connecting β1 to α1 and α1 to β2 are longer in S_{b2} (13 and 10 residues, respectively) than in S_{a1} (5 and 7 residues, respectively). The remainder of the S_{b2} structure encompassing β1, β3, α2, and β4 is very similar to S_{a1}. Heteronuclear NOE dynamics data for S_{b2} were consistent with the NMR structure (**Fig. S9D**). In particular, the relatively long β1-α1, α1-β2, and β2-β3 loops were found to be the most flexible on the ps-ns timescale.

1 Chen, Y. *et al.* Rules for designing protein fold switches and their implications for the folding code. *bioRxiv*, 2021.2005.2018.444643, doi:10.1101/2021.05.18.444643 (2021).
2 He, Y., Chen, Y., Rozak, D. A., Bryan, P. N. & Orban, J. An artificially evolved albumin binding module facilitates chemical shift epitope mapping of GA domain interactions with phylogenetically diverse albumins. *Protein Sci* **16**, 1490-1494 (2007).
3 Alexander, P., Fahnestock, S., Lee, T., Orban, J. & Bryan, P. Thermodynamic analysis of the folding of the Streptococcal Protein G IgG-binding domains B1 and B2: why small proteins tend to have high denaturation temperatures. *Biochemistry* **31**, 3597-3603 (1992).
4 Haglund, E. *et al.* Trimming down a protein structure to its bare foldons: spatial organization of the cooperative unit. *J Biol Chem* **287**, 2731-2738, doi:10.1074/jbc.M111.312447 (2012).
